# Response to Toshihide Tsuda, Yumiko Miyano and Eiji Yamamoto [1]

**DOI:** 10.1186/s12940-022-00952-x

**Published:** 2023-01-26

**Authors:** Joachim Schüz, Evgenia Ostroumova, Ausrele Kesminiene, Louise Davies, Hyeong Sik Ahn, Kayo Togawa, Salvatore Vaccarella

**Affiliations:** 1 International Agency for Research on Cancer, World Health Organisation (IARC/WHO), Lyon, France; 2 Department of Surgery–Otolaryngology–Head and Neck Surgery, The Dartmouth Institute for Health Policy and Clinical Practice, Geisel School of Medicine, Hanover, NH USA; 3 VA Outcomes Group, Department of Veterans Affairs Medical Center, VT White River Junction, USA; 4 Department of Preventive Medicine, College of Medicine, Korea University, Seoul, South Korea; 5 National Cancer Center Institute for Cancer Control, Tokyo, Japan

**Keywords:** Thyroid cancer, Incidence, Overdiagnosis, Nuclear accident

## Abstract

**Background:**

Using a toolkit approach, Tsuda et al. critiqued work carried out by or in collaboration with the International Agency for Research on Cancer (IARC/WHO), including the IARC technical publication No. 46 on “Thyroid health monitoring after nuclear accidents” (TM-NUC), the project on nuclear emergency situations and improvement on medical and health surveillance (SHAMISEN), and the IARC-led work on global thyroid cancer incidence patterns as per IARC core mandate.

**Main body:**

We respond on the criticism of the recommendations of the IARC technical publication No. 46, and of global thyroid cancer incidence evaluation.

**Conclusion:**

After nuclear accidents, overdiagnosis can still happen and must be included in informed decision making when providing a system of optimal help for cases of radiation-induced thyroid cancer, to minimize harm to people by helping them avoid diagnostics and treatment they may not need.

We noted the following publication by Tsuda et al. critiquing work carried out by or in collaboration with the International Agency for Research on Cancer (IARC/WHO) [1], including the IARC technical publication No. 46 on “Thyroid health monitoring after nuclear accidents” (TM-NUC) [2], the project on nuclear emergency situations and improvement on medical and health surveillance (SHAMISEN) [3], and the IARC-led work on global thyroid cancer incidence patterns as per IARC core mandate [4, 5]. For the critique of our work Tsuda et al. used what they refer to as a “toolkit”, the purpose of which is to identify epidemiologic data misused by “powerful interests, particularly those with a financial stake…whose interest are not aligned with the public health sciences”. While Tsuda et al. may disagree with the conclusions reached in the publications, the selected items of the toolkit they apply to criticise it are misapplied – the work by IARC/WHO created no financial benefit for those publishing the work, and the mission of these organizations is explicitly oriented toward public health. We were also concerned to see that the authors confused study designs (page 15 [1]), bringing further misinterpretation of the weight of evidence to their critical evaluation of the papers that they are quoting.

IARC technical publication No. 46 “Thyroid health monitoring after nuclear accidents” is a forward-looking report coordinated by IARC that used scientific evidence and expertise of a large group of international scientists representing a wide spectrum of disciplines. The group was assembled to advise countries how to prepare immediate and long-term response in case of a nuclear power plant accident, with regard to a possible increase in thyroid cancer incidence related to such accident [2]. The report applied the principles of screening, and evidence from thyroid cancer pathology, natural history, epidemiology, diagnostics and clinical management in general population as well in the populations affected by the Chernobyl and Fukushima nuclear accidents [6, 7]. The report’s recommendations are not based on systematic reviews but drawn from all peer-reviewed publications relevant to the project objective. While we wish that our recommendations will never be needed, we believe they represent the recommended best practices for the data currently available.

IARC has been leading work on evaluation of global thyroid cancer incidence which provided convincing scientific evidence of an impact of thyroid care treatment patterns on incidence trends, first for adults [4], and then for children and adolescents [5] (latter not cited by Tsuda et al. [1]). Overdiagnosis in thyroid cancer is a well-recognized phenomena [8]. It globally affects a large number of people and has been acknowledged to result in overtreatment of many otherwise healthy individuals, exposing them to the potential for treatment-related complications and psychological harms, facts reflected in guidelines from the major professional organisations such as the American Thyroid Association (ATA) [9, 10]. The risk of overdiagnosis is also applicable in the setting of nuclear accidents and must be included in informed decision making when providing a system of optimal help for cases of radiation-induced thyroid cancer, to minimize harm to people by helping them avoid diagnostics and treatment they may not need.

## Abbreviations

ATA: American Thyroid Association
IARC: International Agency for Research on Cancer
WHO: World Health Organisation

## References

[1] Tsuda T, Miyano Y, Yamamoto E. Demonstrating the undermining of science and health policy after the Fukushima nuclear accident by applying the toolkit for detecting misused epidemiological methods. Environ Health. 2022. 10.1186/s12940-022-00884-6. 10.1186/s12940-022-00884-6 PMC9400325 36002833

[2] IARC Thyroid Health Monitoring after Nuclear Accidents Expert Group (2018). Thyroid health monitoring after nuclear accidents.

[3] Cléro E, Ostroumova E, Demoury C, Grosche B, Kesminiene A, Liutsko L, et al. Lessons learned from Chernobyl and Fukushima on thyroid cancer screening and recommendations in case of a future nuclear accident. Environ Int. 2021. 10.1016/j.envint.2020.106230. 10.1016/j.envint.2020.106230 33171378

[4] Vaccarella S, Franceschi S, Bray F, Wild C, Plummer M, Dal Maso L. Worldwide thyroid-cancer epidemic? The increase impact of overdiagnosis. N Engl J Med. 2016. 10.1056/NEJMp1604412. 10.1056/NEJMp1604412 27532827

[5] Vaccarella S, Lortet-Tieulent J, Colombet M, Davies L, Stiller C, Schüz J, et al. Global patterns and trends in incidence and mortality of thyroid cancer in children and adolescents: a population-based study. Lancet Diabetes Endocrinol. 2021. 10.1016/S2213-8587(20)30401-0. 10.1016/S2213-8587(20)30401-0 33482107

[6] Thomas G. Radiation and thyroid cancer - an overview. Radiat Prot Dosim. 2018. 10.1093/rpd/ncy146. 10.1093/rpd/ncy146 30165692

[7] Yamashita S, Suzuki S, Suzuki S, Shimura H, Saenko V. Lessons from Fukushima: latest findings of thyroid cancer after the Fukushima nuclear power plant accident. Thyroid. 2018. 10.1089/thy.2017.0283. 10.1089/thy.2017.0283 PMC5770131 28954584

[8] Davies L, Welch HG. Increasing incidence of thyroid cancer in the United States, 1973-2002. JAMA. 2016. 10.1001/jama.295.18.2164. 10.1001/jama.295.18.2164 16684987

[9] Francis G, Waguespack S, Bauer A, Angelos P, Benvenga S, Cerutti J, et al. Management guidelines for children with thyroid nodules. Thyroid. 2015. 10.1089/thy.2014.0460. 10.1089/thy.2014.0460 PMC4854274 25900731

[10] Haugen B, Alexander E, Bible K, Doherty G, Mandel S, Nikiforov Y, et al. American Thyroid Association management guidelines for adult patients with thyroid nodules and differentiated thyroid cancer: the American Thyroid Association guidelines task force on thyroid nodules and differentiated thyroid cancer. Thyroid. 2016. 10.1089/thy.2015.0020. 10.1089/thy.2015.0020 PMC4739132 26462967

